# Address

**Published:** 1873-05

**Authors:** 


### ADDRESS.

### ON BEHALF OF THE GRADUATING CLASS AT THE COMMENCEMENT OF THE OHIO DENTAL COLLEGE, NOV. 5TH, 1873.

Mr. President and Gentlemen of the Faculty :

In the observance of the usual custom of pronouncing the valedictory address upon occasions of this kind, it is the fashion to employ the language of praise and adulation. But, disclaiming all motives to be fashionable, I employ the simple language of sincerity and thankfulness, when I beg to express to you our profound appreciation of your efforts, your perseverance and industry, in promoting us in the knowledge and science of Dentistry ; these qualities, coupled with the knowledge and ability which you have brought to your responsible offices, have indeed made our opportunities rare and valuable. We hope, Gentlemen, that they have not gone unimproved and unappreciated by us.

The relation of preceptor and pupil which we have sustained toward each other for two years, has not been productive of advantage to us in a professional sense alone, but has created, in our daily intercourse, a personal attachment which renders our parting from you painful and reluctant.

Yet, having finished the prescribed course, we go hence, bearing in our Diplomas the evidence of honorable graduation. But we will bear in mind the admonition so often given us, that the *study* of Dentistry does not end here ; that Dentistry is an acknowledged science ; that its study is to be continued through long years of practice ; that a Dentist may and should become learned as well as skillful in his Profession.

And now, Gentlemen, again expressing our thanks for the skill and industry with which you have conducted us through our course of study, and the patience with which you have borne with us—we bid you adieu.

Fellow Graduates :

These closing exercises dissolve our relations as classmates and fellow students of this College.

An almost hourly association, a common end in view, and mutual sympathy, have together engendered among us a brotherly attachment which coming years will strengthen loiterers by the way side.

In the course of our studies we have learned that Dentistry is a science demanding study and reflection as well as skill; that to become experts in the art of Dentistry it is absolutely necessary to extend our knowledge and investigation into the realms of scientific research, for it is truly said that "A principle in science is a rule in art."

Hence, while we are permitted to practice our profession, every sense of professional pride and honor, as well as our personal good, demands that we hold the science of Dentistry above the realm of quacks and pretenders. This can be done only by acquainting ourselves from year to year, with the literature, discoveries and principles of our profession. The value of this professional scholarship is becoming more important as the popular demands upon dental surgery increase and the popular intelligence upon the subject spreads.

We can remember when "tooth drawing" was practiced by the most ignorant and bungling of people. We can remember that in many villages and even in our cities, the barber displayed in front of his shop, a sign inscribed "Bleeding, leeching and tooth drawing;" and to give his surgical profession greater prominence, adopted the original plan of establishing before his door an emblem of his skill in a striped pole, whereon the stripes represented the flesh the bandage and the blood. But the march of common sense has removed the occupation of tooth drawing and surgery from such crude hands, and our tonsorial friend has been compelled to remove the sign of bleeding, leeching and tooth drawing; but pride forbade that every vestige of so honorable an occupation should be obliterated, and so to-day the "striped pole" is the universal sign of the barber's shop, and to him the most unmistakable monument of the lost art.

But Gentlemen, I fear, judging from the way Dentistry is sometimes praticed in this city and elsewhere, that some of our tonsorial friends have made a mistake, and removed the wrong sign. That if, at some of our so called dental establishments the sign that indicates dental surgery were removed, and the "striped pole" substituted, it would indicate a more legitimate business for the operator within.

My friends, it behooves us to avoid the at mosphere of quacks ; to keep before us the model of the perfect dentist.

A discriminating popular intelligence will place every man upon his proper level.

It demands of all a thorough acquaintance with and skill in his occupation.

It is said that there is no science but what abuts upon, and is in some degree interwoven with every other science. I think this is true of Dentistry. I do not undertake to urge that a professional dentist should master all the sciences, but I do say that he should acquaint himself with such principles of every other science as do or may contribute to that which he has chosen as his business in life.

Therefore my fellow Graduates, let us upon this occasion, in taking farewell of our *Alma Mater* and of each other, resolve to walk in the choicest fields of our profession, and not be loiterers by the way side.

